# Cantilever-Based Sensor Utilizing a Diffractive Optical Element with High Sensitivity to Relative Humidity

**DOI:** 10.3390/s21051673

**Published:** 2021-03-01

**Authors:** Catherine Grogan, Faolan Radford McGovern, Rory Staines, George Amarandei, Izabela Naydenova

**Affiliations:** 1 School of Physics and Clinical and Optometric Sciences, Technological University Dublin Grangegorman, D07EWV4 Dublin, Ireland; catherine.grogan@tudublin.ie (C.G.); c16342516@mytudublin.ie (F.R.M.); c15552907@mytudublin.ie (R.S.); 2 Centre for Industrial and Engineering Optics, Technological University Dublin, FOCAS Research Institute, Camden Row, D08 CKP1 Dublin, Ireland

**Keywords:** optical sensors, cantilever sensor, bilayer, holographic sensor, diffractive optical sensor

## Abstract

High-sensitivity and simple, low-cost readout are desirable features for sensors independent of the application area. Micro-cantilever sensors use the deflection induced by the analyte presence to achieve high-sensitivity but possess complex electronic readouts. Current holographic sensors probe the analyte presence by measuring changes in their optical properties, have a simpler low-cost readout, but their sensitivity can be further improved. Here, the two working principles were combined to obtain a new hybrid sensor with enhanced sensitivity. The diffractive element, a holographically patterned thin photopolymer layer, was placed on a polymer (polydimethylsiloxane) layer forming a bi-layer macro-cantilever. The different responses of the layers to analyte presence lead to cantilever deflection. The sensitivity and detection limits were evaluated by measuring the variation in cantilever deflection and diffraction efficiency with relative humidity. It was observed that the sensitivity is tunable by controlling the spatial frequency of the photopolymer gratings and the cantilever thickness. The sensor deflection was also visible to the naked eye, making it a simple, user-friendly device. The hybrid sensor diffraction efficiency response to the target analyte had an increased sensitivity (10-fold when compared with the cantilever or holographic modes operating independently), requiring a minimum upturn in the readout complexity.

## 1. Introduction

Cantilever-based sensors typically are using silicon micro-cantilevers, and they have gained extensive interest due to their small size, mass production and high sensitivity. Early application of silicon cantilevers as sensors began when researchers focused on the fact that silicon cantilevers used in atomic force microscopy (AFM) deflect due to changes in relative humidity (RH) [1]. Micro and nano cantilever-based sensors continue to draw attention, and it has been demonstrated that they are able to detect a variety of biological [2,3,4,5,6,7,8,9], physical [10,11,12], and chemical analytes [13,14,15,16]. The microcantilever-based sensing platform is a label-free detection technique that has been proved as a viable alternative solution to conventional sensing systems, such as the assaying procedures [4]. For example, microcantilever arrays, functionalized on one side with aptamers, were utilized to detect dopamine, a marker for a series of neurological disorders. Such an example demonstrates the vastness of applications in which microcantilever-based sensors can be used. In addition, it also proves their ability to detect low molecular weight molecules at low concentrations [9]. A microcantilever pressure sensor using a resonant sensing chip with microcantilever has been shown to operate by sensing the surrounding gas molecules loading on the sensing surface [14]. This method utilizes the changes measured in the resonant frequency of the microcantilever in response to pressure differences [14]. Microcantilever-based sensors have also been demonstrated as a novel mean of measuring the degradation of biopolymers used as carriers in drug delivery devices [17]. Thus, they have potential applications as biosensors, chemical sensors, portable devices, medical devices and security control [17,18,19,20]. Consequently, the microcantilever-based sensors can offer the opportunity of measuring various analyte species in low concentration but also investigate the fundamental interactions among these species. Characterizing these interactions can be a challenging task to accomplish using other sensing methods, and, therefore, the cantilever sensors can be thought of as being fundamental research devices.

The detection mode of cantilever-based sensors can be divided into two main groups: static and dynamic. The static mode operates by measuring a change in deflection of the cantilever beam due to a change in surface stress of one side compared to the other, thus inducing a deflection similar to the bimetallic effect [21,22,23,24]. The dynamic mode operates by monitoring the resonant frequency of a vibrating cantilever [25,26]. Typically, in the static mode cantilever sensor, only the sensitive layer interacts with the target analyte, while the other layer (or surface) remains inert (or it has a negligible response) to the analyte presence. This interaction changes the surface stress of the “sensing” layer, thus producing a cantilever deflection.

To improve their sensitivity, typically, such sensors require large numbers of units and processing steps controlled through powered interface circuits, the readout being external and performed by expensive equipment. Many efforts have been made to increase the sensitivity of microcantilever-based sensors by patterning to increase the surface area of the sensing layer of the microcantilever. For example, an aluminum substrate was fabricated with an anodic aluminum oxide layer with ordered nanowells to increase the surface area and, thus, the sensitivity of cantilevers used to detect moisture concentration [27,28]. Other work has shown that potentiostatic anodization of the aluminum layer on a microcantilever forms nanoporous alumina without altering the shape, resonance properties or the spring constant of the cantilevers [29].

Many types of microcantilevers as humidity sensors have been reported [30]. Microelectromechanical systems (MEMS) have been demonstrated to detect changes in humidity using the adsorption or desorption of moisture from titanium oxide nanoparticles. The titanium oxide was deposited on a moving plate, which experienced a change in electrical parameters as a result of the adsorption or desorption of moisture [31]. A large humidity range (35–95%) was tested, and the sensor showed a linear detection response, but its sensitivity was rather low [31]. A MEMS mechanical oscillator has been used to measure RH by correlating the change in mass of multilayers of nanomaterials on the oscillator to changes in RH, a resolution of ~1.8% RH being obtained across a range of 0 to 80% RH [32]. Silicon microcantilevers coated with graphene oxide as the sensing layer have also been tested to detect humidity between 10 and 90% RH, but changes in the sensitivity measured in different RH ranges were observed. These systems, like many of the previously identified sensors, utilize more complex electronic and hardware sensor systems, which ultimately increases their cost and thus limits the range of applications [33]. These continuous efforts emphasize that alternatives to silicon cantilever sensors are needed to circumvent the relatively high-cost related to the detection methods and readout complexity. Examples reported in the literature include paper-based cantilever sensors to detect the presence of volatile organic solvents [34] and a piezoresistive layer integrated into a polydimethylsiloxane (PDMS) substrate to measure changes in the mechanical activity of heart cells [35], but the simultaneous enhancement of the sensitivity, cost, and simplicity of use are still remaining a challenge.

Holographic sensors, introduced in [36] and recently reviewed in [37,38], are based on detecting the change in the properties of a holographic optical element as triggered by a change in their environment. Since holographic optical elements are characterized by their ability to diffract light, it is usually their diffraction efficiency or the spectral response of the device that is interrogated [39]. Holographic sensors have the ability to be miniaturized to be compatible with smartphone technologies; they can be tuned to different analytes by modification of the hologram containing polymer layer, are relatively low-cost and compatible with mass production technologies [40,41,42,43,44]. Applications of holographic sensors include volatile organic components, alcohol, metal ions, glucose, pressure, temperature, RH, etc., but in these sensors, the sensitivity remains a challenge and requires further improvements. Holographic sensors are based on the detection of either dimensional changes of the layer (usually a photopolymer) in which the hologram is embedded, a change in the refractive index of the layer or the refractive index modulation in the hologram. It has been previously demonstrated experimentally and modeled theoretically [45,46] that exploring dimensional changes in the holographic layer allows for much higher sensitivity to be achieved. Typically, the holographic sensors are deposited on a substrate that restricts their swelling or shrinkage, in a lateral direction, due to the presence of the analyte. Furthermore, because they are coated on a substrate, all sensors reported in the literature are practically flat layers [36,37,38,39,40,41,42,43,44,45,46]. For example, in [36], a holographic sensor with changing optical properties as the result of a biochemical reaction between the hologram/hologram support medium and the analyte species was introduced. The sensor is based on a reflection hologram recorded in a flat surface layer, and its operation is demonstrated in the presence of a number of analytes among these being the trypsin, concentration of water in toluene, and the content of ethanol in water. Comprehensive reviews of holographic sensors research, methodology for their fabrication, functionalized materials used for detection of the target analytes and the current achievement and limitations are presented in [37,38,39]. Although in some of the reviewed articles, the sensors have microscopically patterned surfaces, they all utilize structures that are flat on macroscopic level. A recently reported work [40] on the development of a glucose sensor by utilizing a metal-free transparent layer, which swells in the presence of the analyte, is another example of the “flat sensitive layer adhered to a substrate” approach. This approach has its benefits, particularly when a smartphone detection of the response of the sensor is considered, as demonstrated in [41]. The authors demonstrate that smartphones have the potential to serve as low-cost point-of-care diagnostic device readers by developing an algorithm with inter-phone repeatability. The algorithm allows a smartphone camera to read semiquantitative tests rapidly with minimal operator intervention and was tested in experiments determining the concentrations of protein, glucose, and pH. A departure from the “sensitive layer adhered to a substrate” approach is reported in [42], in which an increase of the sensitivity of substrate-free flat reflection hologram due to the added freedom of the layer to swell/shrink is demonstrated. Another method of improving the sensitivity of the holographic sensors utilizes diffraction structures created in functionalized micropatterned surface layers [43,44], thus revealing the potential of DOEs in optical sensing. Despite the large number of experimental reports examining the properties of holographic sensors [38,39], the detailed theoretical analysis is relatively limited. Two recent publications focus on transmission surface [45] and volume [46] holographic gratings, which are recorded and operate as macroscopically flat layers, demonstrate that the dimensional change in the layer containing the hologram is the key contributor for achieving high sensitivity; Therefore, a sensor configuration that utilizes an additional degree of freedom of the layer (including deflection), which can enhance its dimensional change as a result of changes in their environment, will bring significant benefits to their sensitivity, as any detuning from the Bragg angle of incidence of the probe beam will be achieved and detected more easily.

The present work introduces a novel concept and studies experimentally a new hybrid optomechanical sensors that have the potential to overcome the issues related to the complex high-cost of the cantilever sensors and can enhance the sensitivity of macroscopically flat holographic sensors. A cantilever, operating in the static mode, with a unique configuration comprising a bilayer sensor with an optically structured analyte responsive layer, is introduced. This hybrid sensor uses a macro-cantilever formed by a polydimethylsiloxane (PDMS) layer as its inert substrate and a photopolymer holographic grating as the sensitive layer. To demonstrate the sensor operation and assess its sensitivity, the RH was monitored over a wide range (10–70% RH). The RH as an analyte was selected for a number of reasons: first—low-cost, highly sensitive sensors to RH are still under development and in demand; second—measuring RH involves using a non-toxic analyte, and it is a suitable model analysis from a proof-of-concept perspective, third—the previous experience in developing flat holographic sensors for RH [47,48] allowed for a direct comparison with the existing holographic sensor technology. Opto-mechanical sensors have been previously explored, for example, sensors based on photoelastic effect [48,49,50,51,52]. The novelty of this work relies on the conversion of a minute mechanical signal into a strong optical signal induced by the response of a holographic diffractive structure. This unique approach to the design of the sensor platform delivers high sensitivity combined with low-cost and miniaturization. This article presents, to the best of the authors’ knowledge, for the first time, a sensor combining the cantilever sensor mechanism with a photopolymer grating able to produce a highly sensitive, simple, and low-cost sensor with a sensitivity that can be tuned by the spatial frequency of the diffraction grating.

## 2. Materials and Methods

### 2.1. Cantilever Deflection and Bragg Selectivity Curve Measurements

**Figure 1:**
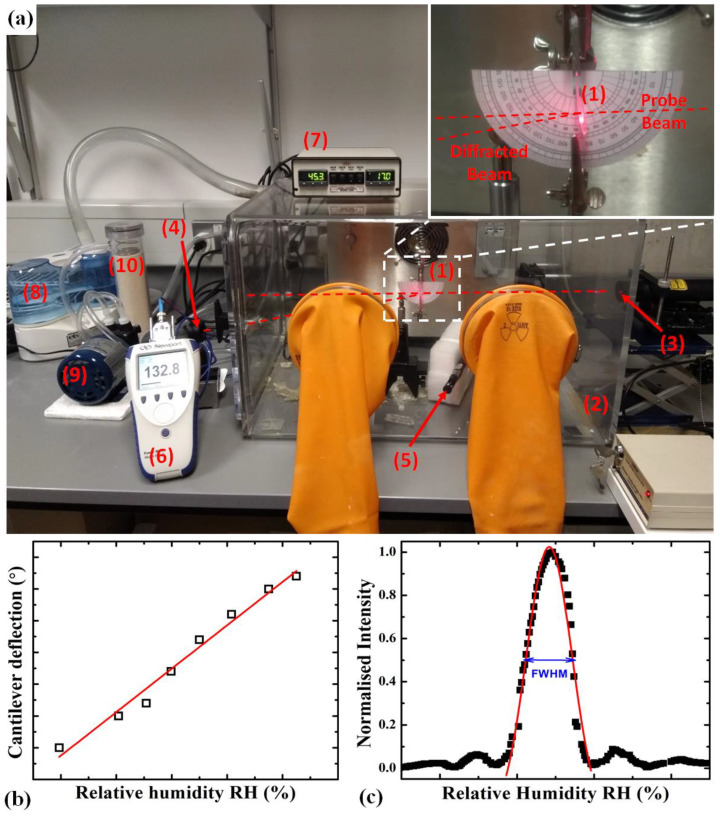
(**a**) Photograph of the experimental setup: the cantilever sensor (1) placed within the humidity and temperature-controlled environmental chamber (2). A probe beam is sent from the laser diode (3), the diffracted beam intensity is measured by the photo-detector (4) connected to the power meter (5). The humidity and the temperature in the chamber were monitored through the sensor (6) and controlled with the microcontroller (7) with the help of the humidifier (8), the pump (9) and the desiccant cartridge (10). Inset: A photograph of the hybrid photopolymer polydimethylsiloxane (PDMS) cantilever sensor system. The typical paths of the probe and diffracted beams are schematically presented as a guide to the eye only. (**b**) Typical cantilever deflection that can be visually evaluated both as angular (in °) deflection as the humidity changes. The angular deflection was then transformed into linear deflection (in mm). (**c**) Typical Bragg diffraction selectivity curve measured as the humidity was varied. A typical Gaussian fitting of the main peak and the full-width half-maximum (FWHM) position are also shown.

In this work, the cantilever beam (manufactured as described in the supplementary material Figure S1 and Section 2.2) was placed vertically, and, on exposure to the target analyte, a change in surface stress caused it to deflect (Figure 1a and the inset). The direction in which the cantilever deflects will depend on the relative position of the two layers in respect to the vertical axis. For example, when viewing the hanging cantilever from the side, the photopolymer layer is to the right, and the PDMS layer is to the left. On increasing RH, the sensing photopolymer layer absorbs moisture and expands, thus inducing a deflection to the left. A decrease in RH causes the photopolymer layer to shrink, thus inducing a deflection to the right. In our experimental configuration, the deflection was to the right as the RH decreases and to the left as the RH increased (Figure 1a inset). By placing a protractor directly behind the cantilever, a deflection angle can be easily measured, thus providing a simple, user-friendly sensor output (Figure 1b). Moreover, additional high sensitivity information can also be obtained from such a configuration by recording the diffraction efficiency of the diffraction optical element (DOE), in this case, a grating (Figure S2a), at a particular deflection/RH, thus obtaining the Bragg selectivity curve (Figure 1c). The change in intensity of a diffracted laser beam at either side of the Bragg angle showed high sensitivity to a small change in analyte (here, RH) concentration. As the cantilever angle changes, the incident angle of the probe beam deviates from the Bragg angle, and the diffraction efficiency can change from 0 to its maximum, with cantilever deflection in the order of 1–2 degrees. Depending on the type of DOE, its efficiency can approach 100%, which means that the incident beam is fully redirected in the direction of the first order of diffraction, and no energy propagates in the original direction. For example, in holographic volume gratings recorded in the photopolymer used in the present study, the maximum diffraction efficiency of 98% has been measured in layers of 75 µm thickness.

The cantilever (as presented in Figure 1) was placed in a humidity and temperature-controlled environmental chamber. The environmental conditions in the chamber were monitored and controlled using an ETS dual control system, Model 5503–11200 (Electro-Tech Systems Inc., Perkasie, PA, USA). The humidity level in the chamber can be reduced down to 10% RH by the desiccant/pump dehumidification system, while an ultrasonic humidification system allows the relative humidity to be increased up to 100%. The heating system can increase the temperature from ambient temperature to 50 °C, the chamber being able to maintain the relative humidity and temperature with an accuracy of ±1% and ±1 °C, respectively. At the heart of the ETS dual control system sits the sensor model 554, which is calibrated and certified temperature-compensated sensor for RH measurements.

The humidity response of the photopolymer/PDMS cantilever sensor was measured by placing the cantilever vertically with the top 5 mm of the cantilever held between two glass slides in the relative humidity-controlled chamber (Figure 1a). The cantilever was placed with the photopolymer layer to the right when viewed from the front of the system. The chamber temperature was maintained at 30 °C. Prior to taking measurements, each cantilever was initially exposed to a temperature of 30 °C for 10 min. This step was carried out to allow the photopolymer layer to stabilize. The cantilever deflection was then recorded (Figure 1b) by visually reading the angle of deflection of the cantilever using a protractor placed directly behind the cantilever (Figure 1a). Cantilevers with no grating and with gratings of various spatial frequency values were investigated. The Bragg selectivity curve for the grating pattern cantilevers was measured using a 633 nm He–Ne laser and a Newport power meter, model 843-R (Newport Spectra-Physics Ltd., Oxfordshire, UK).

The diffractive grating (Figure S2a,b) of the sensor was interrogated by measuring the Bragg selectivity curve (Figure 1c). The deflection of the cantilever sample leads to a change of the incidence angle and, consequently, to a variation in the measured diffraction efficiency, which can be theoretically modeled with the help of Equation (1) [45].

### 2.2. Preparation of Photopolymer Grating Sensing Layer

The photopolymer solution was prepared from 17.5 mL of 10% polyvinyl alcohol, 0.8 g of acrylamide, 0.2 g of *N*,*N*’ methylene bisacrylamide, 2.26 g of triethylamine and 4 mL of erythrosin B. The photopolymer solution (0.6 mL) was drop-casted to coat a microscope slide 25 × 70 mm^2^. After drying for 24 h, the thickness of the photopolymer layers was 60 ± 3 μm. Holographic gratings with either 800 lines/mm or 500 lines/mm (±10 lines/mm) spatial frequencies were recorded on the slides. For both spatial frequencies, a two-beam holographic recording setup with a 1:1 beam ratio was used [53]. The beam ratio was controlled with the help of two half-waveplates and a polarization beam splitter. Each beam had an intensity of 0.5 ± 0.01 mW/cm^2^ for the 500 lines/mm and 0.85 ± 0.01 mW/cm^2^ for the 800 lines/mm. The two intensities were previously optimized for the selected spatial frequency of recording. The wavelength of the recording beams was 532 nm. The diffraction efficiency was measured in real-time during the recording using a 633 nm He–Ne laser. Every grating used had a diffraction efficiency of 60%. At the end of the holographic recording, the slides were bleached with UV light and left in a desiccator for at least 24 h. The sequence of steps involved in the fabrication of the bilayer cantilever is shown in Figure S1.

The PDMS layer was prepared using silicone elastomer (SYLGARD® 184 silicone elastomer kit, Dow Corning, Midland, MI, USA) and its curing agent with a base ratio of 1:10 by weight. The mixture was thoroughly stirred. It was then left to degas in a low vacuum chamber until all the bubbles were removed and the liquid mixture was clear. The resulting PDMS was drop-casted onto the gratings (already cast on the glass slide) and cured for 1.5 h at 65 °C. This secured complete curing of the PDMS independently of its thickness. Cantilever beams (~23 mm (length) × 5 mm (width)) (Figure S2b) were cut and peeled away from the glass slide in order to obtain a self-standing polymer bi-layer cantilever (Figure S2c). The fabricated cantilevers were held at the top between two glass slides, allowing a cantilever of length 17 mm to hang down vertically (Figure 1a). The thickness of the PDMS and, consequently, the overall thickness of the cantilever was varied by controlling the amount of PDMS cast.

### 2.3. Assessment of Sensor Outputs

As previously mentioned, the diffractive grating (Figure 1a inset) of the sensor was interrogated by measuring the Bragg selectivity curve (Figure 1c). The deflection of the sample leads to a change of the incidence angle and variation in the measured diffraction efficiency *η*, which can be theoretically modeled with the help of Equation (1) [45]:

(1) $$\eta =\frac{{\left(\sin{\left(\phi^{2}+\xi^{2}\right)}^{1/2}\right)}^{2}}{1+\frac{\xi^{2}}{\phi^{2}}}$$

where *ϕ* is the phase difference between the probe and the diffracted beam introduced by the diffraction grating and *ξ* is proportional to the deviation from the Bragg angle, Δθ, at which the maximum diffraction efficiency is observed. The *ξ* parameter is proportional to the thickness *d* of the grating and inversely proportional to its period Λ (which determines the spatial frequency of the grating SF, SF = 1000/Λ line/mm). The exact relationship is presented in Equation (2) [45]:

(2) $$\xi =\frac{Kd}{2}\Delta \theta =\frac{\pi d}{\Lambda }\Delta \theta$$

where *K* is the grating vector magnitude.

To demonstrate the dependence of the full-width half-maximum (FWHM) of the Bragg selectivity curve, theoretical curves are presented in Figure S3. First, theoretical curves of the Bragg angular selectivity are simulated utilizing Equation (1) and presented in Figure S3a,c. The dependence of the full-width half-maximum (FWHM) of the Bragg selectivity curves on the spatial frequency (Figure S3b) and the thickness of the grating (Figure S3d) reveal that these two physical parameters of the grating can be utilized to control the sensitivity of the optomechanical sensor. For example, if very high sensitivity is needed, gratings with higher spatial frequency or thickness must be utilized. This would also mean that the operational range covered by a single grating will be limited. In order to cover a larger operational range, more than one diffractive optical element will need to be inscribed in the same location of the photopolymer layer. Alternatively, if a thin low spatial frequency grating is utilized, the operational range will be larger, although with somewhat lower sensitivity.

It is worth noticing that a typical Bragg selectivity curve has two lower intensity satellite peaks. In the proposed sensor transducer, only the signal produced by the central peak is considered. In a further refinement of the sensor platform, an arrangement must be made to suppress the two satellite peaks. This effect is called apodization, and it has been used previously in optical filters [54]. In holographic gratings, this, for example, can be achieved by selecting the recording conditions and by controlling the absorption properties of the photosensitive layer in order to record an apodized grating.

The Bragg angular selectivity curve was obtained by measuring the diffracted beam intensity through the cantilever, while the relative humidity in the chamber and, thus, the deflection of the cantilever was varied. The decrease in the diffracted beam intensity as the cantilever deflected slightly to the right or to the left was measured. As a result, the Bragg angular selectivity curve was produced in response to the decrease and increase in RH.

While the angular deflection was the direct measurement, this was also converted to linear deflection as it can be directly related with the stress measurements through the Stoney equation [55]:

(3) $$\Delta \sigma =\frac{Et^{2}}{3\left(1-v\right)L^{2}}.\Delta z$$

where *L* is the length of the cantilever, v is the Poisson ratio, *E* is Young’s modulus, t is the thickness of the cantilever, and σ is the surface stress generated as a result of the interactions with the cantilever surface. Thus, the change in surface stress induced on the cantilever surface will be a result of the interaction with the target analyte, RH in this case, with the photopolymer layer [55]. A change in RH leads to the expansion/contraction of the photopolymer to a greater extent than in the PDMS and, therefore, induces a deflection in the cantilever, which is directly related to a change in surface stress.

The changes in the slope of the linear deflection and in the FWHM of the Bragg angular selectivity curve as the RH varied were considered representative gauges for monitoring and assessing the sensitivity of the sensor.

The detection limit, *DL*, of the photopolymer cantilever sensors was evaluated using:

(4) $$DL=\;\frac{R}{S}$$

where *R* is the uncertainty in the measured result and *S* is the sensitivity of the measurement.

## 3. Results

**Figure 2:**
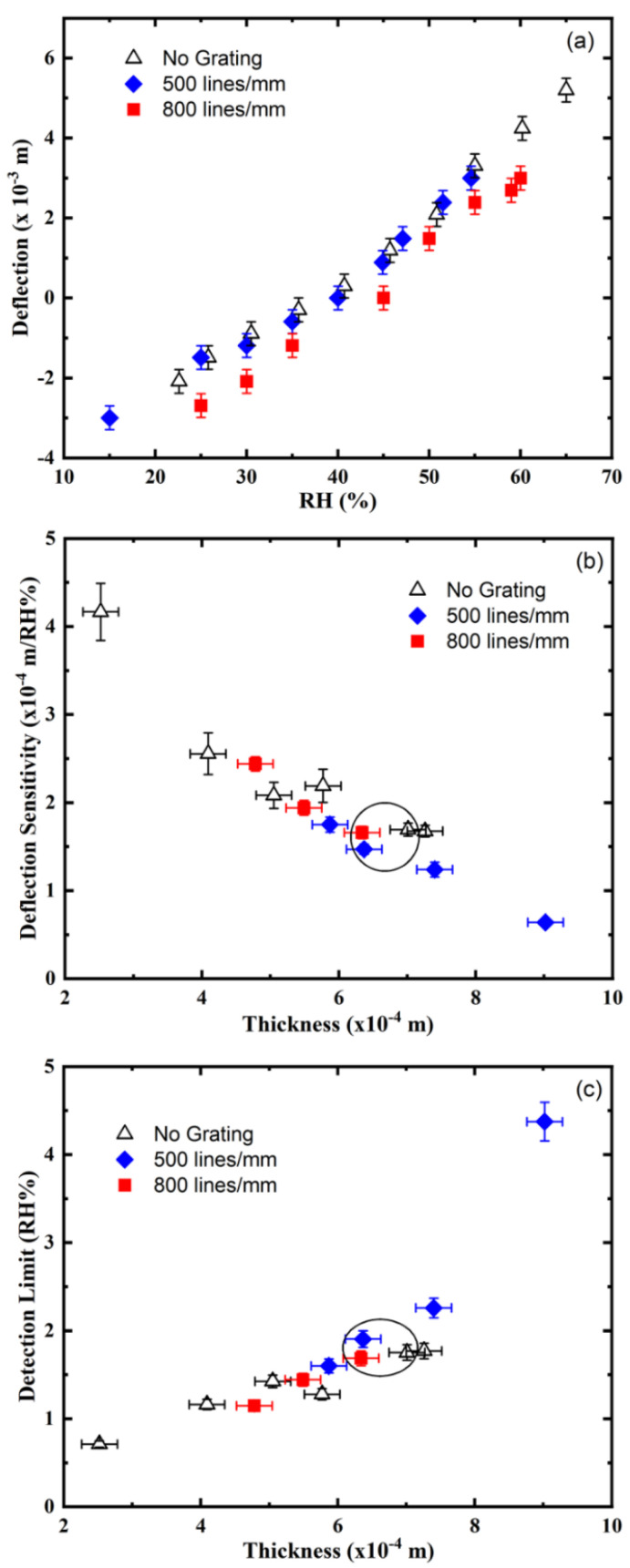
(**a**) Typical cantilever linear deflection as relative humidity (RH) varied for an 800 lines/mm (square), 500 lines/mm (diamond) and a no grating cantilever of similar thickness (637 µm, 634 µm and 701 µm for the 800 lines/m, 500 lines/mm grating and no grating, respectively); (**b**) the deflection sensitivity and (**c**) RH detection limit for two different grating types as cantilever thickness varied. The data for no grating cantilever sensors (black triangle) are also presented. The circled data points show the 3 cantilevers in Figure 2a and the cantilevers investigated using Bragg angular selectivity curves in Figure 3.

Cantilever deflection measurements as the RH varied showed a consistent deflection to the left as the RH increased and a deflection to the right as the RH decreased (Figure 2a). Cantilever sensors of varying thickness for two types of spatial frequency (800 lines/mm and 500 lines/mm) gratings were tested (Figure 2a,b). A close to linear deflection response can be observed on all samples independent of their thickness (see Figure 2a for typical responses). To check the operation of the cantilever sensor without the use of the grating in the photopolymer layer, a cantilever containing a PDMS substrate and a photopolymer layer without a grating was also tested.

Cantilever sensors with no grating showed a similar linear deflection response to changes in RH. At this range of spatial frequency, the pattern recorded in the photopolymer has no significant effect on the deflection sensitivity of the cantilever (see Figure 2b).

The validation at this stage of sensor development was carried out by using the response to RH from a deflection calibration curve of 800 lines/mm cantilever. The relative humidity in the chamber was preset at a given relative humidity and was measured in parallel by the temperature compensated commercial sensor and by the novel cantilever sensor. We observed a maximum of 5.4% deviation of the RH values determined by the two sensors.

The detection limit of RH using the linear deflection output of the cantilever sensors was determined (Figure 2c). Thus, Figure 2c shows a linear increase in detection limit as the cantilever thickness increases up to 800 μm. Above this thickness, the sensitivity of the cantilever sensor decreases significantly, as reflected by the increase in the detection limit as well (Figure 2c). A detection limit of ~1.2% RH was the lowest detection limit measured using the thinnest pattern sample (478 μm) with 800 lines/mm spatial frequency grating, and a 0.71% RH was obtained for a no grating sample of a thickness (252 μm). To fully understand this result, one must consider that the cantilever sensitivity is affected by a series of factors, such as the overall cantilever thickness, the thickness of the individual layers, the grating presence and the interactions between the layers and with the analyte.

**Figure 3:**
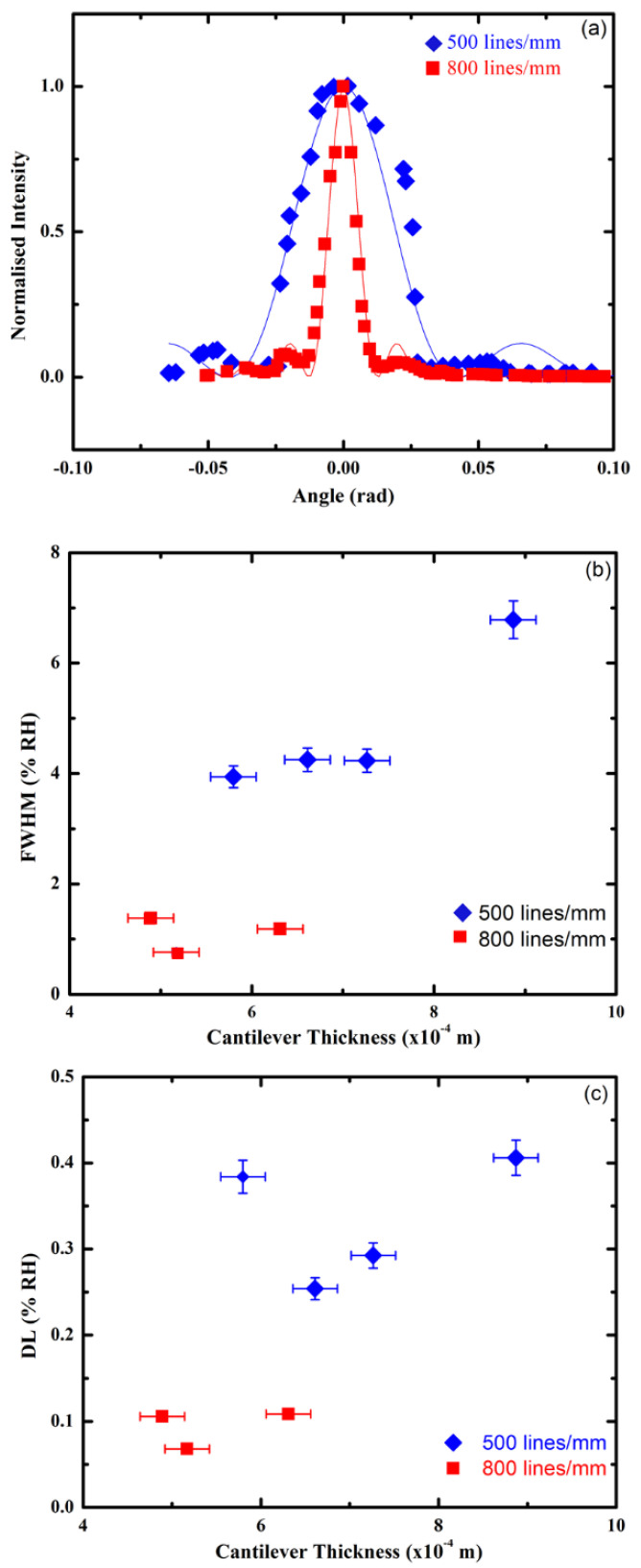
(**a**) Typical Bragg selectivity curves for two samples with a similar thickness (637 µm and 634 µm) and different gratings (800 and 500 lines/mm, respectively). The intensities are normalized in order to allow direct comparison between samples. The fitting was performed using the diffraction efficiency equation (Equation (1)); (**b**) FWHM; and (**c**) the detection limit variation for a cantilever with thickness for a different diffraction grating.

The sensitivity of cantilever sensors was also evaluated using Bragg angular selectivity curves as these could lead to increased sensitivities. Therefore, the Bragg angular selectivity curves were measured as the thickness and spatial frequency of the sensor were changed. The Bragg selectivity curves of two cantilevers of similar thickness with different spatial frequency measured (Figure 3a) show a clear difference in the FWHM values. The cantilever sensor with the 800 lines/mm grating exhibits a significantly narrower FWHM compared to the 500 lines/mm grating. The smaller FWHM measured for the 800 lines/mm samples indicates greater sensitivity to changes in RH for the larger spatial frequency in the photopolymer sensing layer.

The Bragg selectivity curves of all cantilever samples were fitted to a theoretically predicted variation of the diffraction efficiency with a change in incidence angle, given in Equation (1). An example of this procedure can be seen in Figure 3a for the cantilevers with similar thicknesses and different spatial frequency. The R-squared value of the fit was measured to be of the order of 0.9 in both cases, indicating the trustworthiness of the Bragg selectivity curve measurements. However, despite the large value of R and the appropriate fitting of the main peak, the satellites are not always fitted properly (Figure 3a). Therefore, the FWHM of each cantilever sensor was measured from a Gauss fit of the central peak of the Bragg angular selectivity curves (see Figure 1c). A narrower FWHM was observed for all the 800 lines/mm samples compared to the 500 lines/mm samples (Figure 3b). A distinct grouping of the 800 lines/mm and 500 lines/mm independent of cantilever thickness was observed. This distinct grouping of the higher spatial frequency samples is further evidence of the increase in sensitivity, suggesting that the sensitivity can be fine-tuned in these novel sensors using the spatial frequency of the photopolymer grating. Small or negligible changes in the FWHM of each of the grating types, namely 500 and 800 lines/mm, were observed as the thickness changed. This is believed to be due to the small change in deflection angle required for the cantilever to pass through the full Bragg selectivity curve. Therefore, further investigations will be required to be performed over wider ranges of thickness of cantilever samples, but this is not within the purpose of this study.

The detection limit of the cantilever sensors, using the sensitivity of the linear region of the Bragg selectivity curve, was also calculated for all cantilever samples prepared (Figure 3c). The value for resolution in the measurement, R, in this situation was deemed to be conservatively 0.1, which corresponds to a 10% change in diffraction efficiency. The value for intensity was normalized, and the value for *S* was the slope of the linear region of the Gaussian fit to the Bragg selectivity data. The lowest detection limit using the Bragg selectivity measurements was found to be close to 0.1% RH, which is a 10-fold increase in sensitivity compared to that found using the linear deflection method (see Figure 2c).

The 800 lines/mm and the 500 lines/mm grating cantilevers of similar thicknesses also showed different magnitudes of cantilever deflection response for the same decrease in RH (5% RH), with the 800 lines/mm producing a deflection of 1.2 mm to the left and the 500 lines/mm sample producing a 0.8 mm deflection to the left.

The detection limit DL using the Bragg selectivity for the 800 lines/mm spatial frequency showed the best sensitivity at 0.07% RH, while for the cantilevers patterned with the 500 lines/mm grating, the best sensitivity measured was 0.34% RH. Figure 3c also shows that the detection limit can be changed by varying the spatial frequency in the grating. In addition to the spectral frequency, the sensitivity and the DL will be influenced by the complex interplay among the thicknesses of the layers, the overall cantilever thickness, the adhesion properties of the two layers, the Poisson ratio etc. The high sensitivities observed are currently limited to a relatively small operational range, but this range can be further expanded by using a complex diffractive optical element, e.g., multiplexed gratings. Therefore, further experimental and theoretical studies will be needed to elucidate this complex dependency and the influence of each contributing factor.

## 4. Discussion

The experimental results described in the previous section aim to evaluate the feasibility of increasing the sensitivity of a bi-layer cantilever sensor. It is proposed that the incorporation of an optical diffraction element (a volume phase holographic diffraction grating) in one of the layers and optical probing of its diffraction efficiency will provide much more sensitive detection of the changes in the device environment. To the authors’ knowledge, this is the first time when such an optomechanical sensor is reported.

The sensitivity of the cantilever sensor is first evaluated by measuring the angular deflection of the cantilever, as shown in Figure 1. The effect of the spatial frequency of the holographic grating and the thickness of the cantilever on the linear deflection, deflection sensitivity and limit of detection are presented in Figure 2. The linear deflection results in Figure 2a reveal a quasi-linear dependence on the% relative humidity for the studied humidity range (15–65% RH) for three sets of cantilever sensors with similar total thicknesses and 60 μm thicknesses of the photopolymer layer. The three sets of cantilevers differ in the spatial frequency of the grating recorded in their photopolymer layers-0 lines/mm (no grating), 500 lines/mm (2 μm period grating) and 800 lines/mm (1.25 μm period grating), respectively. No significant effect of the spatial frequency of the holographic grating on the mechanical properties of the cantilevers was observed in the linear deflection versus % RH in the studied range of spatial frequencies. The linear deflections for the cantilever without a grating and the one with 500 lines /mm grating are overlapping, while the linear deflection for the cantilever with %RH for the 800 lines /mm is in their immediate lower proximity, with a curve that is parallel to them (Figure 2a, red squares). To further investigate if the observed slight differences are due to variation in the thickness of the cantilevers, we studied a range of cantilevers with different thicknesses and estimated the deflection sensitivities for all three types of cantilevers (with no grating and 500 and 800 lines/mm spatial frequency gratings). As seen from Figure 2b, the linear sensitivity for these sets has a linear dependence on the thickness of the cantilevers, and there is no significant difference introduced by the spatial frequency of the grating. The detection sensitivity is seen to increase with the decrease of the cantilever thickness. This can be explained by the fact that the photopolymer layer experiencing the dimensional change remains at a constant thickness of 60 μm. When the overall cantilever thickness is decreased, a force with a similar magnitude is applied to a thinner second layer, and, consequently, a larger deflection is observed. Thus, this experiment clearly establishes that one approach to increase the sensitivity is to decrease the overall thickness of the cantilever (with the lowest detection limit achieved being 0.7% RH for cantilevers of 250 μm thickness). Further control over the linear deflection sensitivity can be achieved by understanding better the impact of the adhesion between the two layers and finding a method to modify it, for example, by controlling the roughness of the contact surfaces of the layers, as well as independently controlling their mechanical properties. The mechanical properties can be modified by selecting different holographic recording regimes of polymerization of the photosensitive layers or by varying the concentration of the crosslinker, which will produce polymer chains of different lengths, entanglement and density.

After establishing the detection limits of the sensor by measuring its linear deflection during exposure to different relative humidity, the same cantilevers are interrogated optically by measuring the diffraction efficiency of the incorporated gratings in one of their layers. The high sensitivity of the diffraction efficiency of the holographic grating to any deviation of the incident beam angle from the Bragg angle is utilized in this case. As the relative humidity in the environmental chamber changes, the cantilever is deflected, and, thus, the angle of incidence of the incoming probe beam changes (as presented in Figure 1). The change in diffraction efficiency with the deviation of the incident beam angle from the Bragg angle is measured, and the results are presented in Figure 3a. As a characteristic of the properties of holographic gratings, the FWHM of the Bragg angular selectivity curve is wider for the grating with the lower spatial frequency [38]. The FWHM for the cantilevers with different thicknesses (Figure 3b) demonstrates that there is little influence of the thickness on the FWHM as far as the spatial frequency is kept constant, and this is to be expected since the thickness of the layer containing the grating remains constant. The only exception in this trend is the data point for the cantilever with the largest thickness. This deviation most likely is observed due to the fact that the relationship between the angle of deflection and the relative humidity in the chamber may not obey the same linear dependence as at lower thicknesses. The achieved limit of detection is significantly lower than the one achieved by measuring angular deflection (Figure 3c), thus demonstrating the higher sensitivity of the device when probed optically. The detection limit is much smaller for gratings with higher spatial frequency. This is to be expected since the angular Bragg selectivity curves become narrower with an increase of the spatial frequency (Figure 3a), which is also in agreement with the theoretical prediction (Figure S3). Within the studied range of thicknesses for the two sets of cantilevers, the dependence of the detection limit on the cantilever thickness is similar, and there is a minimum/optimum detection limit observed at a higher thickness for the lower spatial frequency grating. The shape of the detection limit curves implies that there are multiple factors influencing the response of the sensor. Further studies will be needed to identify all factors and find a systematic approach to their control.

In this study, to demonstrate the capability of the proposed sensor platform, the humidity was chosen and, in fact, its relative value being the one to be monitored. Humidity was selected as water vapors are non-toxic, and the temperature and relative humidity controlled environments are readily available. Table S1 compares the achieved limit of detection of the relative humidity of the proposed sensor platform (0.1% RH) to other relevant sensor technologies, such as microcantilevers (1.8% RH) [32], flat holographic sensors (1% RH) [39,47,48] and optical fiber sensors (0.12% RH) [56] previously reported in the literature.

The comparison reveals the high potential of this novel optomechanical sensor. In addition to the high sensitivity, when the low electronics complexity and cost, the low susceptibility to interference from electromagnetic fields, the low– to -medium manufacturing complexity, and the low cost of the sensor transducer are considered, it is clear that this novel sensor technology [57] is compared favorably with optical fiber Bragg grating sensors [16,35,58,59,60] plasmonics/nanoplasmonics sensors [33] and MEMS/NEMS sensors [14,16,30,31,32,33,34,35] and its further development is well justified.

## 5. Conclusions

A unique hybrid cantilever-based sensor using PDMS as an inert substrate and a holographic grating recorded in a photopolymer layer as the sensing layer has been introduced for the first time, and the device enhanced capability of detecting the chosen target of interest, namely the relative humidity RH, was demonstrated in this study. These first results emphasized that the sensitivity can be fine-tuned by controlling the thickness of the cantilever substrate and the spatial frequency of the photopolymer layer.

The lowest detection limit using the Bragg selectivity measurements was found to be 0.07% RH. Increased sensor sensitivity was observed in all cantilevers using the larger spatial frequency grating. These high sensitivities observed are currently limited to a relatively small operational range, and a further increase in the operational range will be targeted by utilizing more complex diffractive structures.

Future work will investigate a wider range of thicknesses, spatial frequencies and polymer bi-layers to provide a better understanding of their influence on sensitivity and cantilever sensor response to the presence of various analytes. The effect of modifying the surface relief properties of the sensing layer on the sensor properties will also be investigated. Evaluation of the stress acting in this system will be considered, and the existence of the pattern in the sensing layer must be accounted for in the theoretical evaluations. Therefore, it is expected that the Stoney equation will be modified, and factors, such as porosity, layers adhesion, etc., must also be considered along with the presence of the pattern. Therefore, such studies have the potential to provide new insights into the mechanical properties of the cantilever and allow for further development of the hybrid cantilever sensors and their implementation in real-world applications.

The holographic photopolymer cantilever sensor outlined in this paper presents a sensor with a dual ability: (1) a simple, easy-to-use visual sensor output (deflection) and (2) a highly sensitive output using an optical readout based on changes in diffraction efficiency. Holographic and microcantilever sensors have already been studied for the detection of volatile organic compounds [37]. It is expected that the hybrid cantilever sensor presented here could provide high sensitivity at the low cost required for indoor air quality control. For example, this can be achieved by monitoring the presence of harmful volatile organic compounds [61] that are in some cases emitted by carpets, paints, furniture, etc.

## Data Availability

Data are not available yet.

## Supplementary Materials

The following are available online at https://www.mdpi.com/1424-8220/21/5/1673/s1, Figure S1: Experimental steps in cantilever preparation, Figure S2: Cantilever sensor description, Figure S3: Theoretical Bragg selectivity curves, Table S1: A comparison of the limit of detection for relative humidity of the proposed technology to other relevant technologies.
