# Supplementary material for: Cantilever-Based Sensor Utilizing a Diffractive Optical Element with High Sensitivity to Relative Humidity
Source: Sensors (Basel). 2021 Mar 1;21(5):1673. doi: 10.3390/s21051673 (PMC7957689; doi:10.3390/s21051673)
Supplement: Supplementary file 1 [file sensors-21-01673-s001.pdf]

Supplementary Material

Cantilever-based sensor utilizing a diffractive optical element with high sensitivity to relative humidity

Catherine Grogan [1], Faolan Radford McGovern [1,2], Rory Staines [1,2], George Amarandei [1*] and Izabela Naydenova [1,2,*]

[1]School of Physics and Clinical and Optometric Sciences, Technological University Dublin, City Center Campus, Dublin, Ireland

[2]Centre for Industrial and Engineering Optics, Technological University Dublin, City Center Campus, Dublin, Ireland

*Correspondence: george.amarandei@tudublin.ie and izabela.naydenova@tudublin.ie

S.2. Experimental details

S.2.1. Experimental steps in cantilever preparation

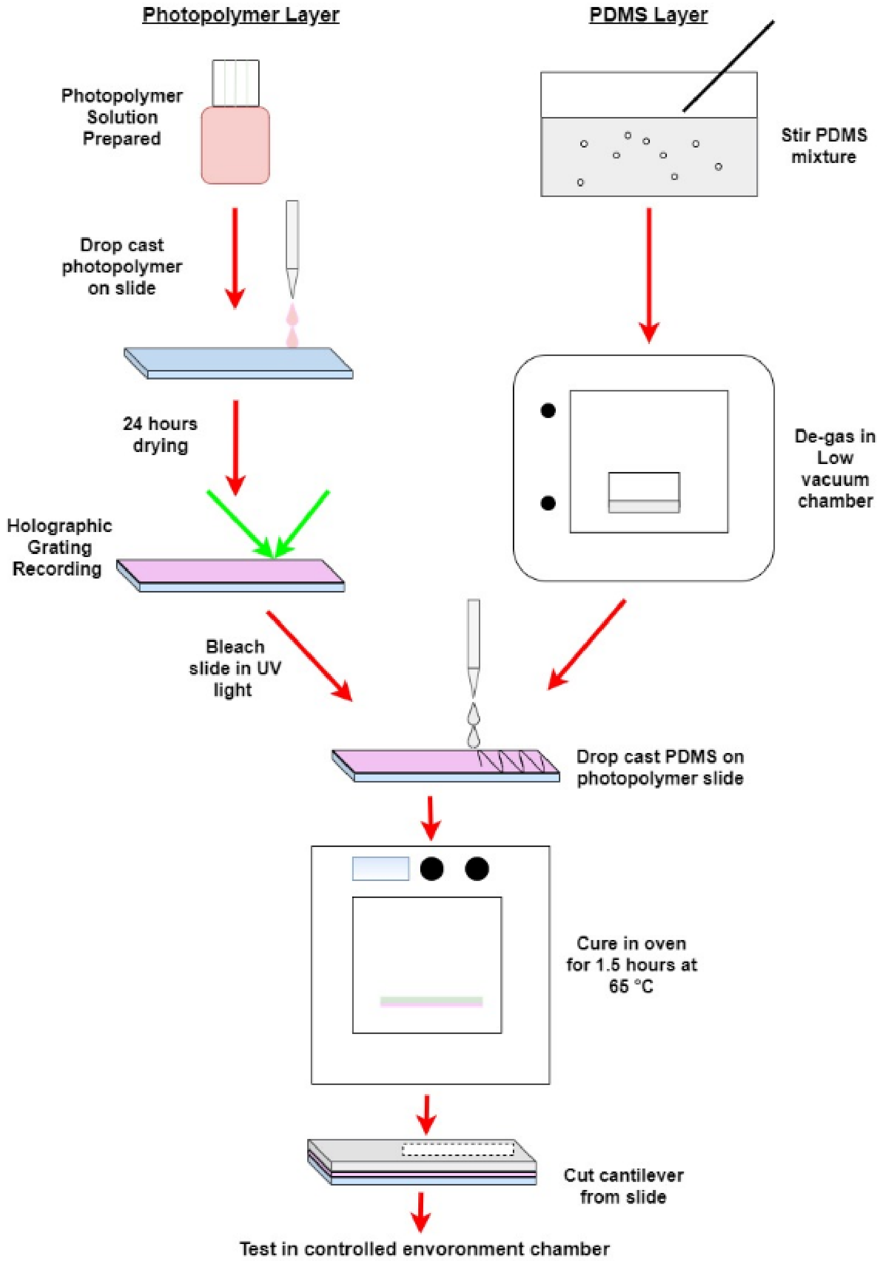

Figure S1. The experimental steps used in this study to prepare the cantilever sensor. The schematic includes the steps involving the preparation of the polydimethylsiloxane (PDMS) solution, the photopolymer layer, the grating recording and the curing stages as described in Materials and Methods section of the paper.

S.2.2. Cantilever sensor description

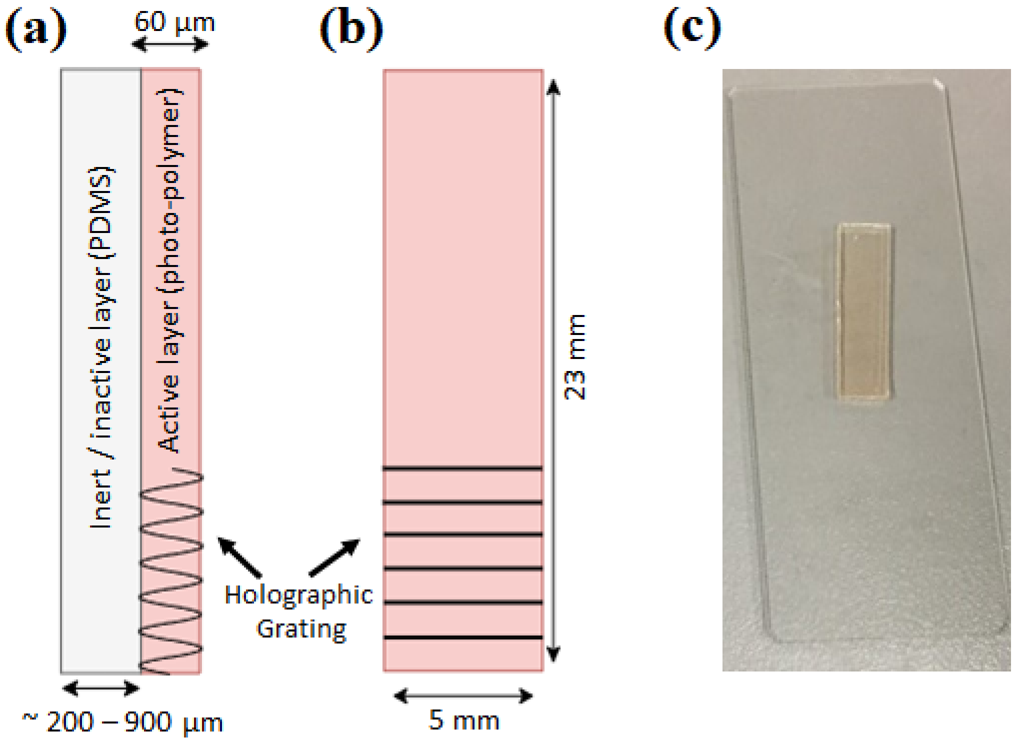

Figure S2. Side (a) and front (b) schematic (not at scale) view of a cantilever sensor formed from an inactive (here, PDMS) layer and an active (here, a photopolymer layer patterned with a diffraction grating) layer. The dimensions and the position of the grating are included. (c) A typical photograph of such bi-layer hybrid cantilever placed on a glass slide.

S.2.3. Theoretical Bragg selectivity curves

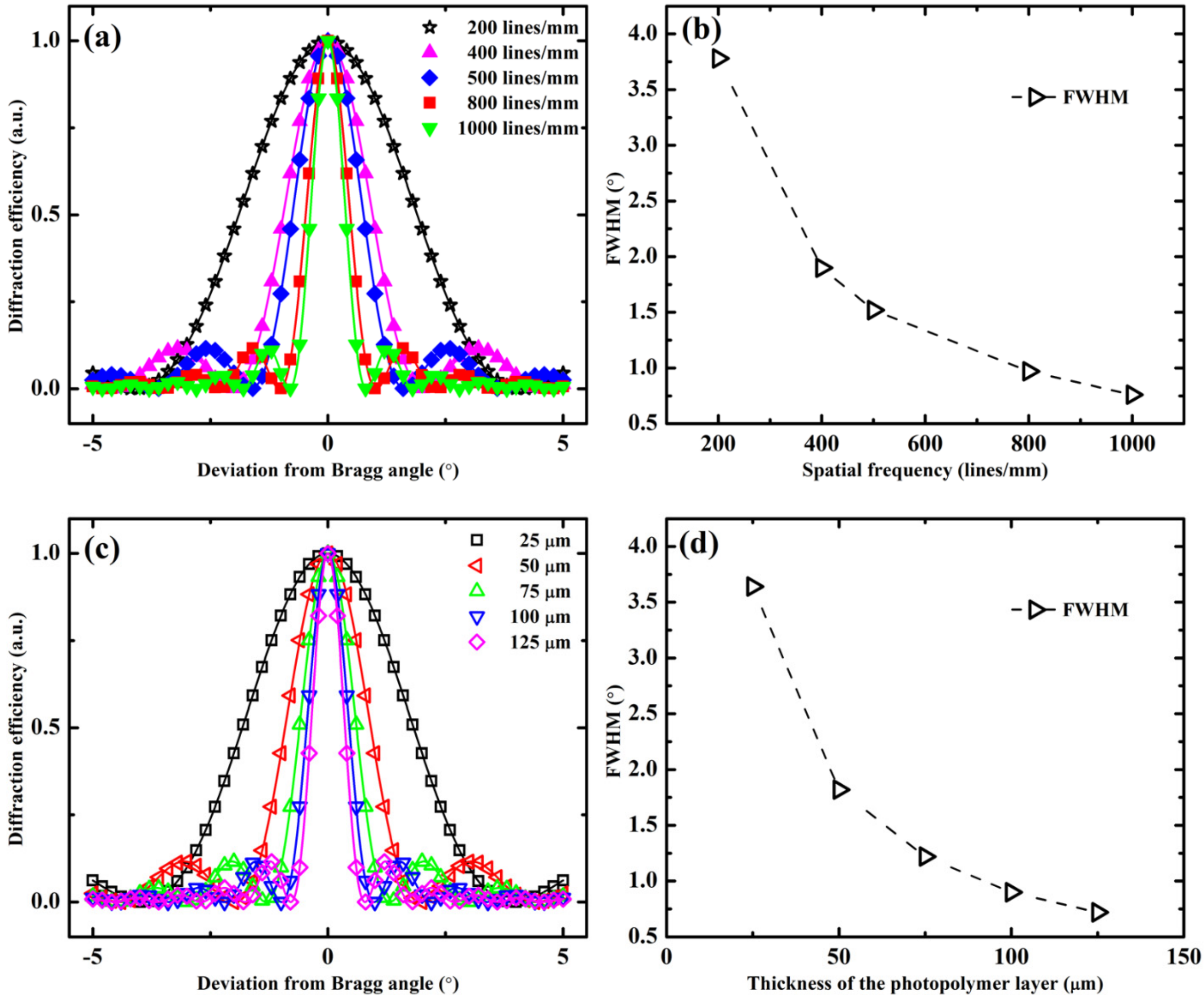

Figure S3. Theoretical curves of (a)diffraction efficiency of holographic gratings with spatial frequency of 200, 400, 500, 800 and 1000 lines/mm and constant thickness of 60 μm; (b) dependence of the FWHM (°) calculated from (a) on the grating spatial frequency; (c) diffraction efficiency of holographic gratings with special frequency of 500 lines/mm and thicknesses of 25, 50, 75, 100 and 125 μm; (d) dependence of the FWHM (°) calculated from (c) on the grating thickness.

S3. A comparison between the new hybrid sensor and other technologies currently available from limit of detection perspective.

Table S1. A comparison of the limit of detection for relative humidity of the proposed technology to other relevant optical and micro-cantilever based technologies.

Sensor technology	Limit of detection	Reference
DOE-cantilevers	0.1 % RH	This paper
Flat holographic sensors	1 % RH	[47], [48], [39]
MEMS	1.8% RH	[32]
Fiber-sensor	0.12% RH	[56]
